# Supplementary material for: netDx: interpretable patient classification using integrated patient similarity networks
Source: Mol Syst Biol. 2019 Mar 19;15(3):e8497. doi: 10.15252/msb.20188497 (PMC6423721; doi:10.15252/msb.20188497)
Supplement: Supplementary file 1 — Appendix [file MSB-15-e8497-s001.pdf]

Appendix

netDx: Interpretable patient classification using integrated patient similarity networks

Authors:
Shraddha Pai[1,2], Shirley Hui[1], Ruth Isserlin[1], Muhammad A Shah[1], Hussam Kaka[1], Gary D. Bader*[1,3,4,5]

Affiliations:
1. The Donnelly Centre, University of Toronto, Toronto, Canada
2. Affiliate Scientist, The Centre for Addiction and Mental Health, Toronto, Canada
3. Department of Molecular Genetics, University of Toronto, Toronto, Canada
4. Department of Computer Science, University of Toronto, Toronto, Canada
5. The Lunenfeld-Tanenbaum Research Institute, Mount Sinai Hospital, Toronto, Canada
* gary.bader@utoronto.ca

Supplementary Figures 3

Supplementary Tables 8

References 12

Supplementary Figures

Appendix Figure S1. Variation in univariate filtering by lasso regression.

Appendix Figure S2. Variation in feature-level scores with increasing number of train/test splits.

Appendix Figure S3. Comparison of netDx and Gene Set Enrichment Analysis for expression-based binary LumA prediction in breast cancer.

Appendix Figure S4. Comparison of selected features from netDx and DIABLO binary breast tumour classifier using RNA and miRNA data.

Supplementary Tables

Appendix Table S1. Comparison of predictor methods for netDx and other methods (PanCancer Survival)

Appendix Table S2. Comparison of netDx performance to PanCancer Survival project broken down by machine-learning algorithm. Bold indicates best AUROC value or significant p-value.

Appendix Table S3. Mean AUROC values reproduced from the PanCancer Survival project.

Appendix Table S4. netDx scores for pathway-level features in asthma case/control prediction. Score shown is the best achieved by a given network for over 70% of the 100 trials. Only networks scoring a max of three or more out of 10 in over 70% trials are shown here.

Supplementary Figures

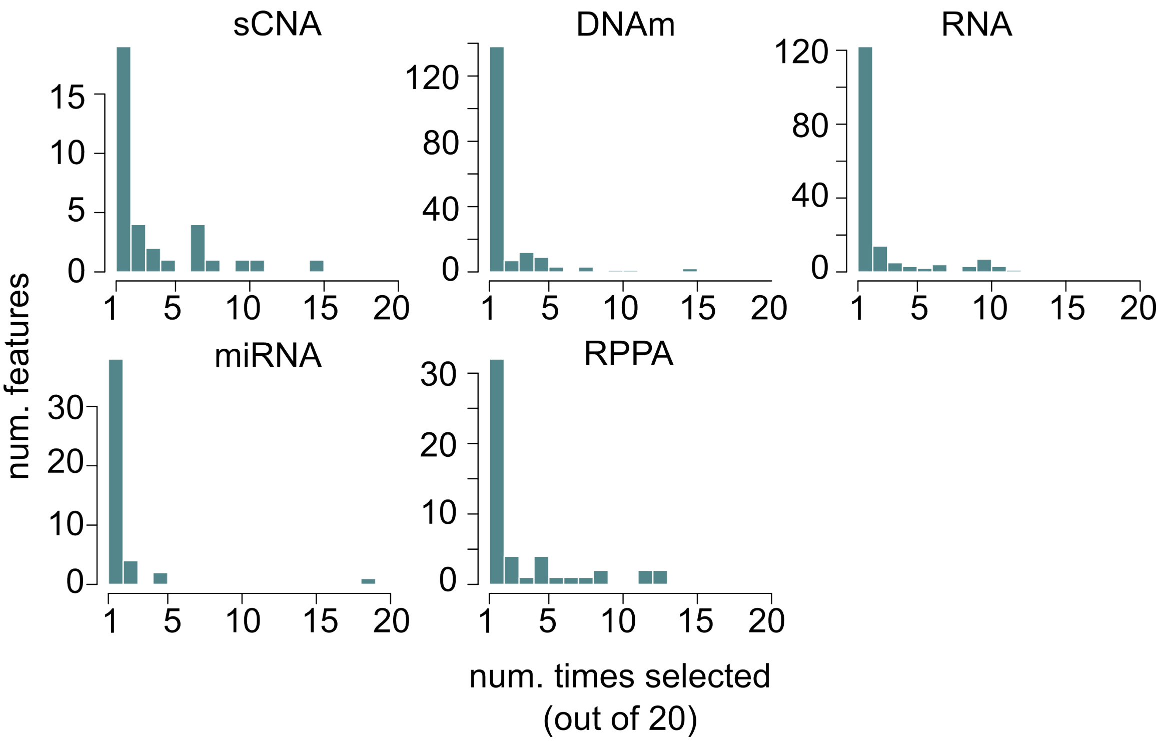

Appendix Figure S1. Variation in univariate filtering by lasso regression. Each panel shows the frequency with which – out of 20 train/splits – a given measure (e.g. transcript for RNA, or protein for RPPA) had a non-zero weight. Data are shown for ovarian cancer survival prediction. The predictor was run for 20 train/test splits. Within each split, lasso regression was run on training samples only (i.e. within cross-validation), and only variables with non-zero weights were used to create patient similarity networks. The x-axis starts at 1. The percentage of variables that never passed lasso regression was: sCNA: 68.8% ; DNAm: 99.3%; mRNA: 99.1% ; miRNA: 94.4% ; RPPA: 72.1%.

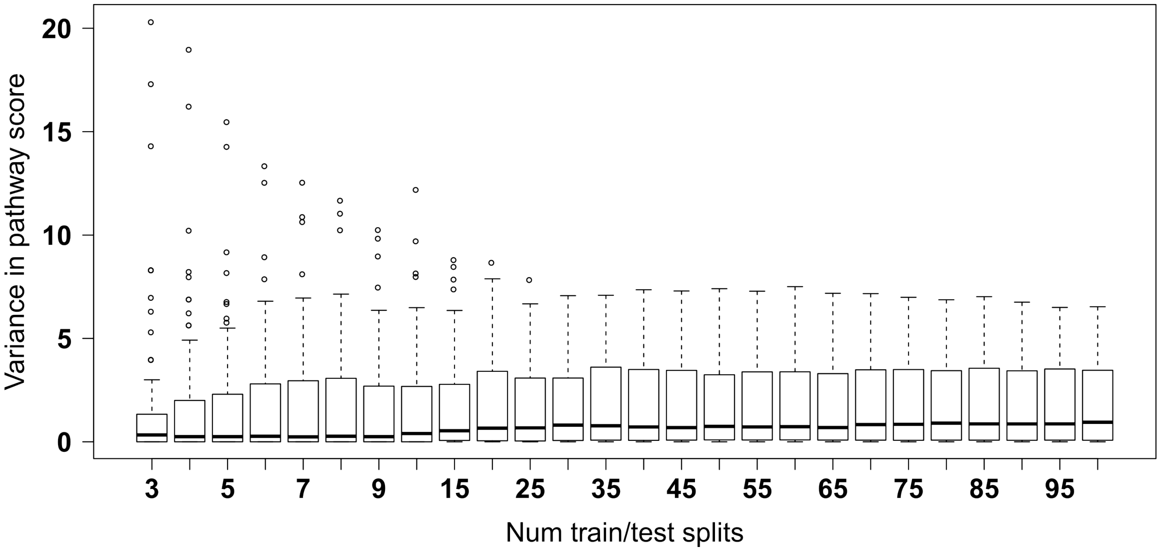

Appendix Figure S2. Variation in feature-level scores with increasing number of train/test splits. The plot shows variance (σ^2) in pathway-level score (out of 10) for the Luminal A ("LumA") class, for gene-expression based binary classification of breast tumours. Each boxplot shows data for a different cumulative number of train/test splits; e.g. the boxplot at x=15 shows pathway-level variance for 15 train/test splits.

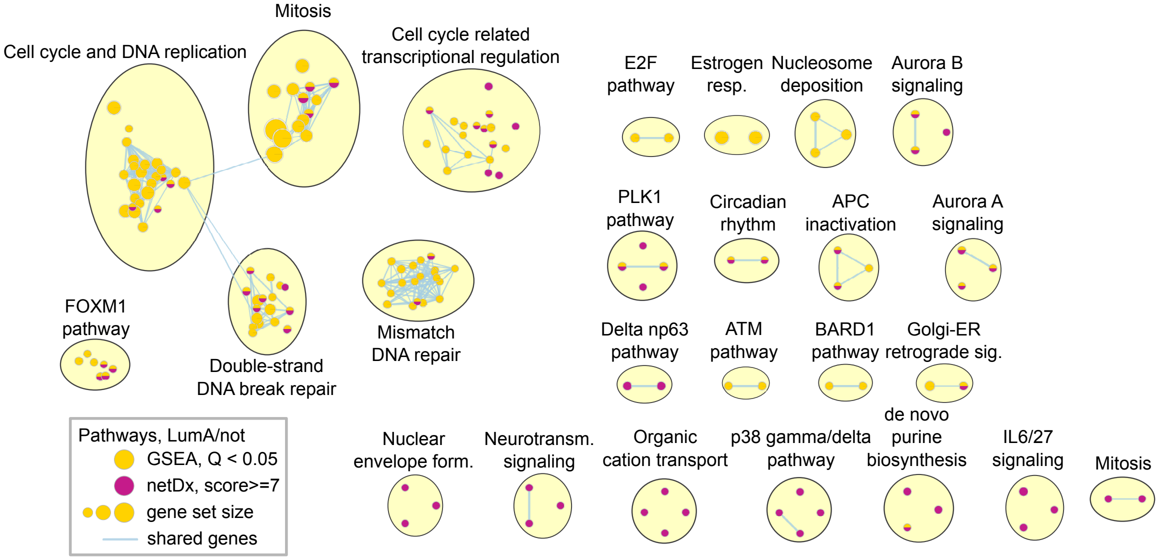

Appendix Figure S3. Comparison of netDx and Gene Set Enrichment Analysis for expression-based binary LumA prediction in breast cancer.

In the enrichment map shown, nodes indicate pathways, and edges indicate shared genes. Node fill indicates whether a pathway was significant in the GSEA analysis (yellow, Q <= 0.05, N=126 pathways), was consistently high-scoring in netDx (magenta; scores>=7 out of 10 in >=70% of 100 splits, N=80 pathways), or both (split fill). Node size represents gene set size. Nodes were connected if they share 40% or more of genes in their gene sets (similarity). Singleton nodes (i.e. nodes not connected to any other nodes) were moved into related clusters if they were found to be connected to at least one node in that cluster in a map with a lower (50%) gene set similarity threshold; other singleton nodes are listed in the full set of pathways in Dataset EV4). The EnrichmentMap app in Cytoscape was used to generate the map (Merico et al., 2011), and the AutoAnnotate app was used to cluster pathways and thematically label clusters (Kucera et al., 2016).

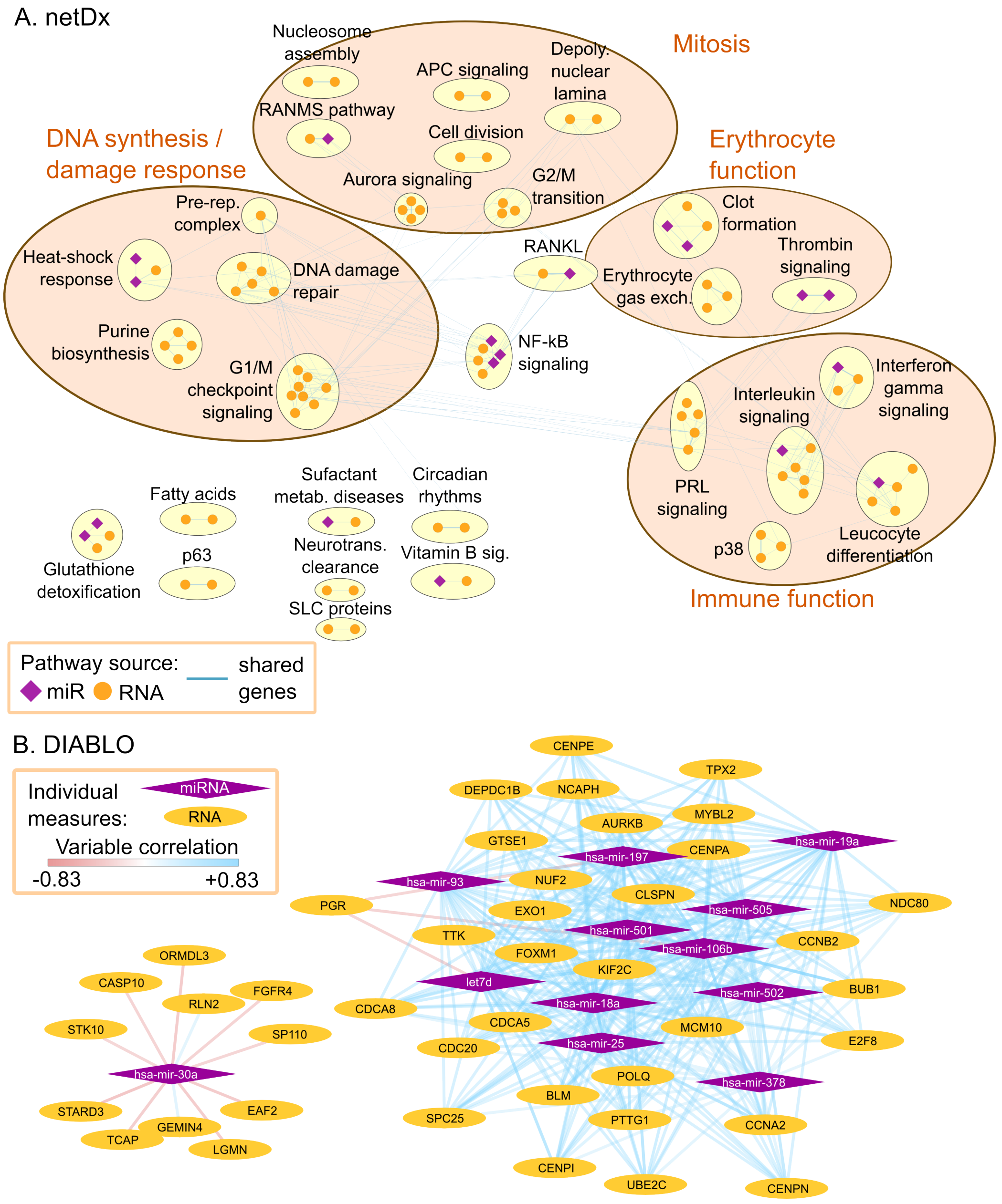

Appendix Figure S4. Comparison of selected features from netDx and DIABLO binary breast tumour classifier using RNA and miRNA data.

A. Features scoring 10 out of 10 in a single train/test split of netDx. Nodes indicate pathway-level features, and edges connect nodes with shared genes. miR-based pathways are shown as purple diamonds, and RNA-based pathways are shown as orange diamonds. To allow related miR and RNA-based pathways to be connected in the network, the corresponding genes – and not miRNA products – were used for the miRNA pathway nodes. EnrichmentMap (Jaccard of 0.05) and AutoAnnotate apps were used to generate the initial map and thematically cluster pathway nodes. Related themes were then manually grouped (tan

circles) and labelled. Singleton nodes (nodes not connected to others) are not shown but are listed with all feature-selected pathways in Dataset EV6.

B. Relevance network from DIABLO showing correlation between individual feature-selected variables for the same prediction task. Nodes represent individual genes (orange ellipses) or miRNA (purple diamonds); edges indicate positive (blue) or negative (red) pairwise correlation.

Supplementary Tables

**A. Variable recoding**		
**Tumour type**	**Yuan et al. workflow (URLs link to R code showing coding)**	**netDx workflow**
**GBM**	Coding: Gender: Female=1, Male=0.	Identical coding
	https://www.synapse.org/#!Synapse:syn1895895 ; main.R	
**KIRC**	Coding: Coding: Grade: {G1,GX} -> G2	Identical coding
	https://www.synapse.org/#!Synapse:syn1895901	
**LUSC**	Coding: Coding: Stage: IA or 1B => I; IIA or IIB => II; IIIA or IIIB => III	Identical coding
	https://www.synapse.org/#!Synapse:syn1895966 ;main.R	
**OV**	Coding: None	Identical coding
	https://www.synapse.org/#!Synapse:syn1895992 ; main.R	
**B. Within cross-validation loop of predictor**		
**Univariate filtering**	Within train/test framework, i.e. applied to training samples before feature selection	Identical except uses lasso regression and keeps variables with non-zero weights
	ANOVA (similar to netDx) or shrinking centroids -> keep top X variables (X=1 to 4 for clinical variables; X = 10-50 for 'omic data). ** Each of these models was separately tested and the best reported in main results. (Partek)	
**Imputation**	Within train/test framework, i.e. applied to training samples.	Identical except uses only imputation by median. Imputation was applied only to GBM
	Imputation by median (continuous variables), by mode (categorical variable) (Partek)	Imputation by median

Appendix Supplementary Table 1. Comparison of predictor methods for netDx and PanCancer Survival.

Method	Median AUROC, other method	Median AUROC, netDx	Num datapoints, other	Num datapoints, netDx	WMW (1-sided) pval
SVM	0.64	**0.67**	40	40	0.17
NC	0.655	**0.67**	40	40	0.05
KNN	0.62	**0.67**	40	40	**0.01**
RF	0.615	**0.67**	40	40	**0.01**
PLS	0.64	**0.67**	40	40	**0.03**
LR	0.63	**0.67**	40	40	**0.01**
DA	0.64	**0.67**	40	40	**0.03**
DDA	0.62	**0.67**	40	40	**0.02**

Appendix Supplementary Table 2. Comparison of netDx performance to PanCancer Survival project, the latter separated by machine-learning algorithm. Bold indicates best AUROC value or significant p-value.

A. Ovarian cancer (OV)

	clin	scna	meth	mRNA	miRNA	prot	Clin SCNA	Clin meth	Clin mRNA	Clin miRNA	Clin Prot
**SVM**	0.62	0.62	0.62	0.63	0.63	0.63	0.64	0.65	0.65	0.64	0.68
**RF**	0.55	0.61	0.58	0.62	0.59	0.55	0.63	0.60	0.63	0.61	0.62
**PLS**	0.65	0.56	0.60	0.64	0.61	0.59	0.64	0.65	0.62	0.64	0.61
**NC**	0.65	0.59	0.58	0.61	0.58	0.60	0.65	0.64	0.66	0.66	0.66
**LR**	0.51	0.58	0.60	0.60	0.59	0.62	0.58	0.64	0.62	0.63	0.65
**DA**	0.65	0.56	0.60	0.64	0.61	0.58	0.64	0.65	0.62	0.64	0.64
**KNN**	0.59	0.59	0.57	0.62	0.58	0.60	0.61	0.59	0.62	0.64	0.65
**DDA**	0.65	0.61	0.60	0.62	0.59	0.58	0.62	0.61	0.62	0.64	0.63

B. Lung cancer (LUSC)

	clin	scna	mRNA	miRNA	prot	Clin SCNA	Clin mRNA	Clin miRNA	Clin Prot
**SVM**	0.56	0.63	0.66	0.53	0.70	0.62	0.67	0.54	0.84
**RF**	0.56	0.57	0.63	0.48	0.64	0.59	0.65	0.49	0.65
**PLS**	0.65	0.57	0.67	0.52	0.62	0.62	0.67	0.55	0.69
**NC**	0.65	0.55	0.62	0.49	0.67	0.63	0.62	0.51	0.71
**LR**	0.51	0.66	0.67	0.51	0.65	0.60	0.67	0.51	0.65
**DA**	0.65	0.57	0.67	0.52	0.62	0.62	0.67	0.55	0.69
**KNN**	0.58	0.62	0.61	0.55	0.64	0.63	0.61	0.55	0.61
**DDA**	0.68	0.57	0.66	0.54	0.67	0.61	0.66	0.54	0.68

C. Glioblastoma (GBM)

	clin	scna	methy	mRNA	miRNA	Clin SCNA	Clin methy	Clin mRNA	Clin miRNA
**SVM**	0.63	0.50	0.59	0.61	0.56	0.67	0.64	0.71	0.64
**RF**	0.65	0.48	0.57	0.57	0.56	0.60	0.59	0.61	0.62
**PLS**	0.67	0.49	0.53	0.59	0.53	0.54	0.58	0.65	0.63
**NC**	0.67	0.49	0.53	0.59	0.54	0.66	0.66	0.67	0.67
**LR**	0.68	0.48	0.54	0.59	0.56	0.56	0.59	0.64	0.63
**DA**	0.67	0.49	0.53	0.59	0.53	0.54	0.58	0.65	0.63
**KNN**	0.64	0.52	0.59	0.58	0.54	0.63	0.64	0.67	0.65
**DDA**	0.67	0.46	0.54	0.57	0.57	0.53	0.56	0.61	0.61

D. Kidney cancer (KIRC)

	clin	scna	meth	mRNA	miRNA	prot	Clin SCNA	Clin meth	Clin mRNA	Clin miRNA	Clin Prot
**SVM**	0.74	0.60	0.71	0.73	0.62	0.72	0.75	0.76	0.73	0.70	0.78
**RF**	0.74	0.55	0.71	0.73	0.69	0.66	0.72	0.75	0.75	0.75	0.72
**PLS**	0.75	0.57	0.69	0.71	0.68	0.67	0.75	0.75	0.74	0.75	0.75
**NC**	0.75	0.60	0.71	0.67	0.67	0.71	0.76	0.75	0.67	0.76	0.76
**LR**	0.69	0.57	0.71	0.70	0.66	0.65	0.69	0.75	0.75	0.73	0.75
**DA**	0.75	0.57	0.69	0.71	0.68	0.67	0.75	0.75	0.74	0.75	0.75
**KNN**	0.74	0.53	0.70	0.72	0.57	0.68	0.72	0.76	0.72	0.69	0.76
**DDA**	0.75	0.59	0.72	0.67	0.68	0.71	0.71	0.74	0.69	0.75	0.73

Appendix Supplementary Table 3. Mean AUROC values from the PanCancer Survival project. Reproduced from (Yuan et al., 2014).

**A. Asthma cases**	
**Feature name**	**max score**
BIOCARTA SET PATHWAY	10
BIOCARTA CTL PATHWAY	9
BIOCARTA D4GDI PATHWAY	9
NOTCH2 INTRACELLULAR DOMAIN REGULATES TRANSCRIPTION	9
SA CASPASE CASCADE	8

**B. Controls**	
**Feature name**	**max score**
BIOCARTA CTL PATHWAY	10
BIOCARTA D4GDI PATHWAY	10
BIOCARTA SET PATHWAY	10
SA CASPASE CASCADE	10
ACTIVATION OF THE MRNA UPON BINDING OF THE CAP-BINDING COMPLEX AND EIFS, AND SUBSEQUENT BINDING TO 43S	8
BIOCARTA DNAFRAGMENT PATHWAY	8
DISEASES ASSOCIATED WITH VISUAL TRANSDUCTION	8
RETINOID CYCLE DISEASE EVENTS	8

Appendix Supplementary Table 4. netDx scores for pathway-level features in asthma case/control prediction

References

Kucera M, Isserlin R, Arkhangorodsky A, Bader GD (2016) AutoAnnotate: A Cytoscape app for summarizing networks with semantic annotations. *F1000Research* **5:** 1717

Merico D, Isserlin R, Bader GD (2011) Visualizing gene-set enrichment results using the Cytoscape plug-in enrichment map. *Methods Mol Biol* **781:** 257-277

Yuan Y, Van Allen EM, Omberg L, Wagle N, Amin-Mansour A, Sokolov A, Byers LA, Xu Y, Hess KR, Diao L, Han L, Huang X, Lawrence MS, Weinstein JN, Stuart JM, Mills GB, Garraway LA, Margolin AA, Getz G, Liang H (2014) Assessing the clinical utility of cancer genomic and proteomic data across tumor types. *Nat Biotechnol* **32:** 644-652
