# Supplementary material for: netDx: interpretable patient classification using integrated patient similarity networks
Source: Mol Syst Biol. 2019 Mar 19;15(3):e8497. doi: 10.15252/msb.20188497 (PMC6423721; doi:10.15252/msb.20188497)
Supplement: Supplementary file 2 — Expanded View Figures PDF [file MSB-15-e8497-s002.pdf]

Expanded View Figures

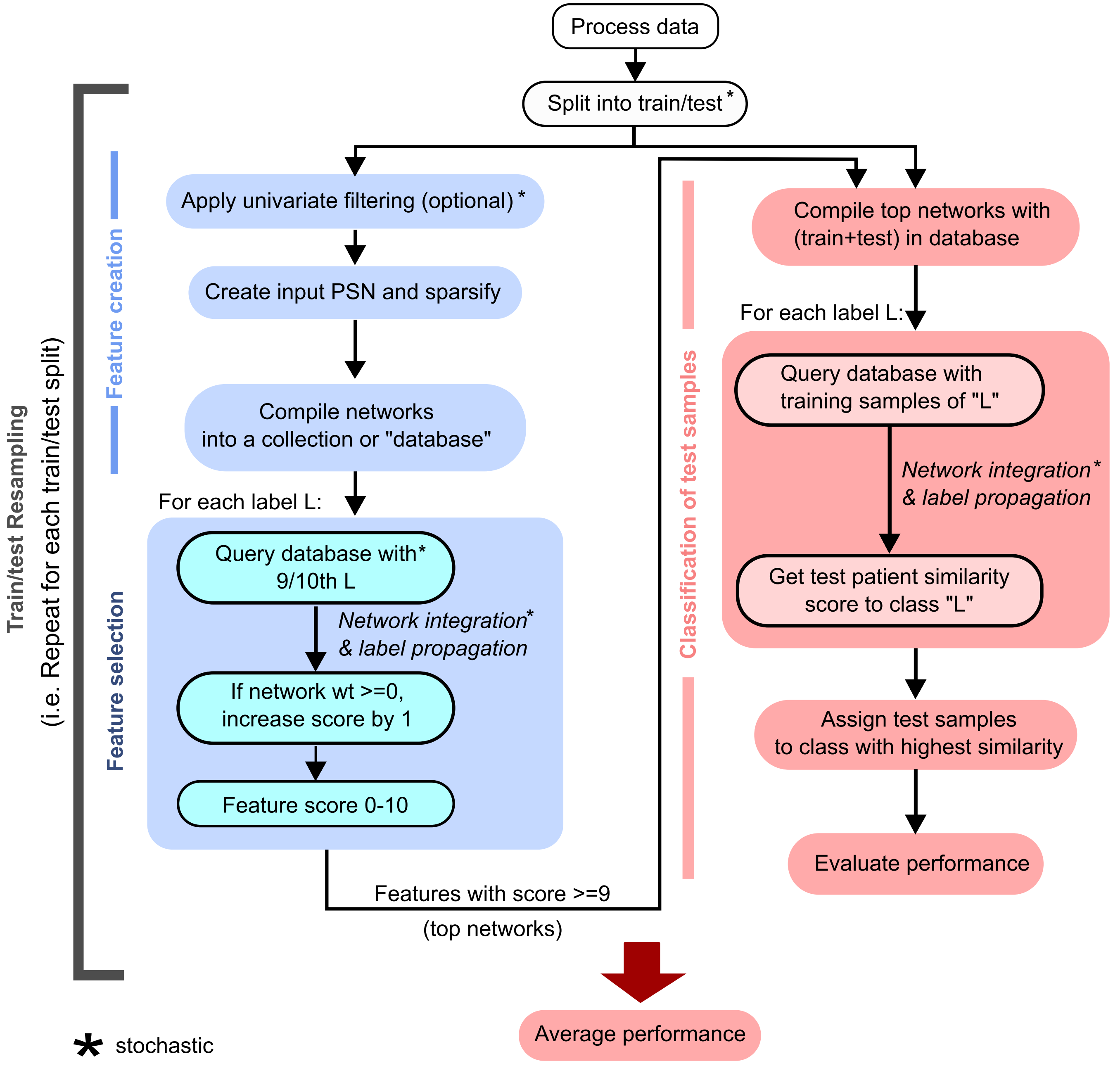

Figure EV1. Detailed workflow of netDx predictor, indicating stochastic components.

Feature selection is shown on the left and uses only training samples (blue boxes). Top-scoring features are used to score the held-out test set (red boxes). Input networks are created and loaded into a GeneMANIA database. Feature selection is achieved by running 10 GeneMANIA queries, once per patient label or category. Note that GeneMANIA queries are run twice. They are first run on a database containing only training subjects and used to compute feature scores. The second time, they are run to assign labels to blind test samples (right). In this instance, the database contains both training and test samples, and is limited to feature-selected networks. Stochastic components of the model are indicated by a black asterisk.

© 2019 The Authors

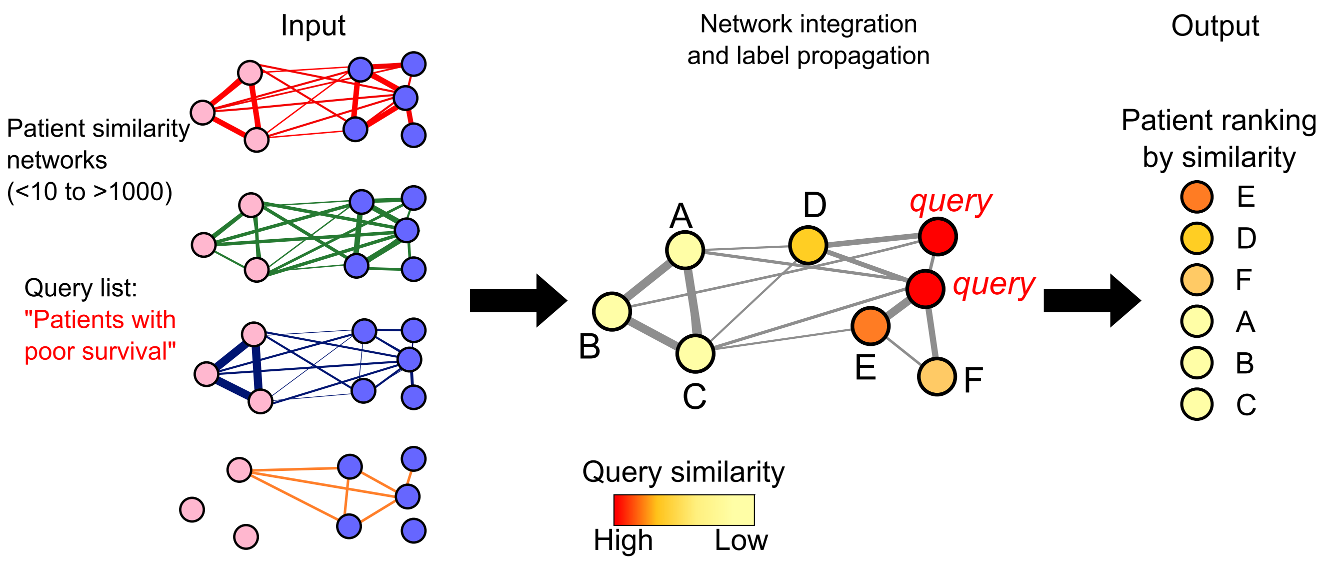

Figure EV2. Conceptual overview of the GeneMANIA algorithm, used by netDx for network integration.

GeneMANIA is a network-based recommender system that ranks all nodes by similarity to an input query (or "positive" nodes). In netDx, the nodes are patients and GeneMANIA uses the set of input patient similarity networks (left). The patient ranking is achieved by a two-step process. First, input networks are integrated into a single association network via regularized regression that maximizes connectivity between nodes with the same label and reduces connectivity to other nodes (middle); this step computes network weights corresponding to predictive value for each network. Second, label propagation is applied to the integrated network starting with the query nodes (red), thereby ranking patients from most to least similar to the query (right).

© 2019 The Authors

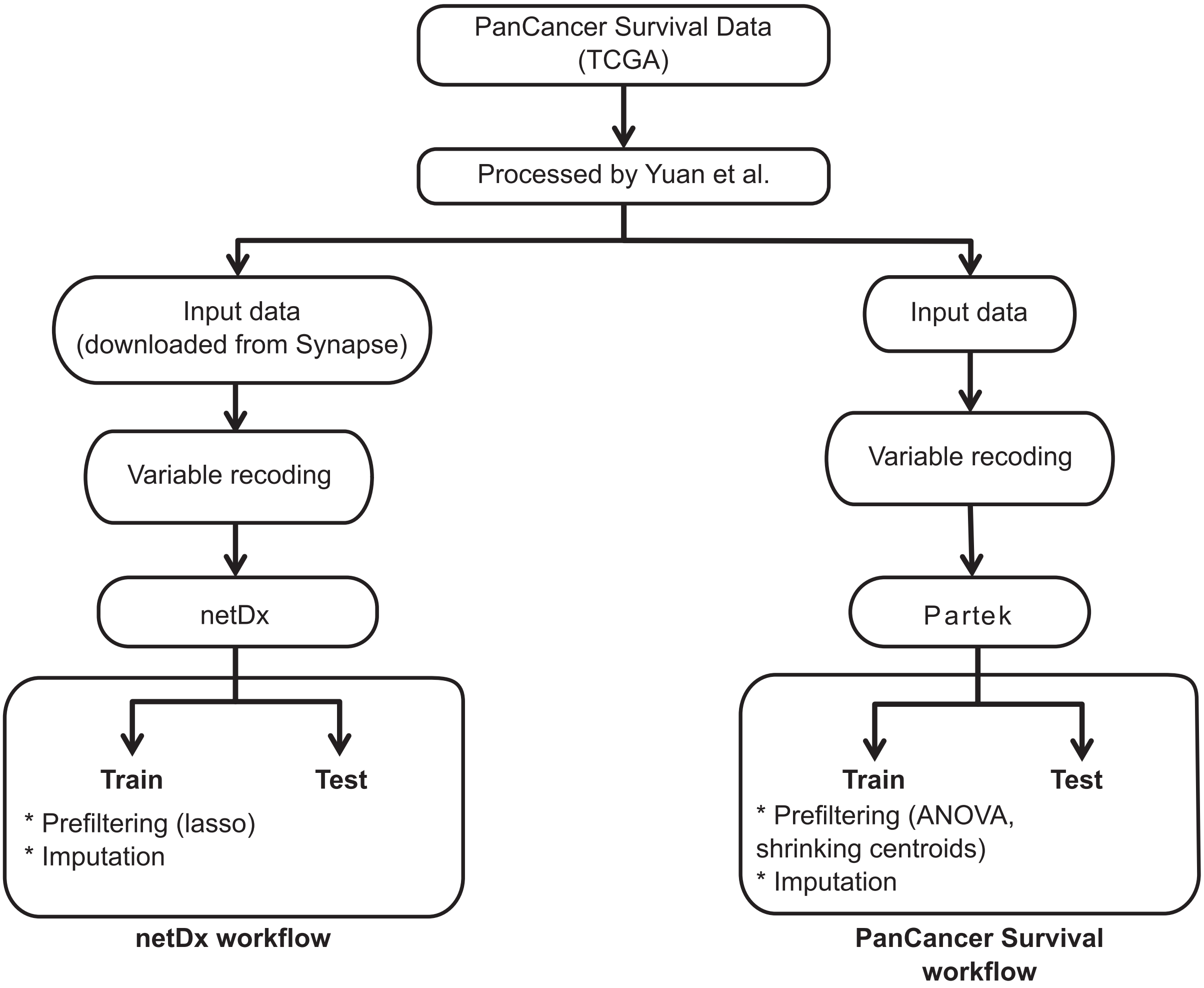

Figure EV3. Comparison of workflow for netDx and PanCancer Survival for benchmarking.

The netDx workflow is shown on the left and PanCancer Survival on the right. To compare netDx to other methods used by the PanCancer Survival project, we downloaded processed data from Synapse (see Data Availability section). To keep all steps except the predictor identical to the PanCancer Survival project, clinical variables were coded identically to the PanCancer Survival project (Appendix Table S1). Processed variables were then provided to the predictor. Univariate prefiltering was performed within the cross-validation framework of the respective predictor (Appendix Table S1); the variability across this process is shown in Appendix Fig S1.

 © 2019 The Authors
